# Functional Molecules in Locally-Adapted Crops: The Case Study of Tomatoes, Onions, and Sweet Cherry Fruits From Tuscany in Italy

**DOI:** 10.3389/fpls.2018.01983

**Published:** 2019-01-15

**Authors:** Roberto Berni, Marco Romi, Claudio Cantini, Jean-Francois Hausman, Gea Guerriero, Giampiero Cai

**Affiliations:** ^1^Department of Life Sciences, University of Siena, Siena, Italy; ^2^Trees and Timber Institute-National Research Council of Italy (CNR-IVALSA), Follonica, Italy; ^3^Environmental Research and Innovation Department, Luxembourg Institute of Science and Technology, Belvaux, Luxembourg

**Keywords:** bioactive molecules, Tuscany, autochthonous ancient varieties, nutraceutics, functional food

## Abstract

The human diet is characterized by highly energetic molecules, but it also requires non-energetic compounds that are equally useful for cell functioning and for preserving the organism's health status. These “functional” molecules are represented by a wide variety of plant secondary metabolites, such as terpenoids, vitamins and polyphenols with antioxidant power. Widespread commercial crop varieties often contain scarce levels of functional molecules, because they have been mostly selected for productivity, rather than for the content of secondary metabolites. Different scenarios (global economic situation, foreseeable environmental changes) are pushing farmers to review the use of high yield crops and to focus on the valorization of locally-adapted plants. This renewed interest is strengthened by the growing need of consumers for functional foods with beneficial effects on human health and by the willingness to promote sustainable low-input agricultural practices exploiting local climate, soil, water, and (micro)biota. Here, we want to discuss a specific case study concerning locally-adapted crops in Tuscany (Italy). Analyses of nutraceutical molecules in locally-grown crop varieties (namely tomatoes, sweet cherries and onions) have shown that they are characterized by substantially higher functional molecule contents than commercial varieties. Our goal is to promote the high-throughput study of locally-adapted varieties to understand, in a medium-term perspective, whether the cultivation of such plants is a valuable support for the diet and an adequate local economic resource. Such plants can provide a boost to the regional economy, by diversifying the local crop-market landscape. Moreover, the exploitation of locally-grown plants results in the manufacture of fully-traceable products (from the raw bioresource to the finished product) with a “0 km” concept that minimizes the C footprint.

## Functional Molecules in the Diet and Their Effects On Human Health

Plant functional molecules (a.k.a as bioactives or health-promoting compounds) are metabolites produced through the plant secondary metabolism that exert biological effects on humans (Azmir et al., 2013; Berni et al., 2018a). Their role in health protection is one of the most important discoveries of the last decade in the biological, medical, and pharmacological fields. The most noteworthy feature of these health-beneficial molecules is their widespread occurrence in fruits and vegetables. This implies the possibility of assimilating them through the daily diet, thereby avoiding any chemical or industrial process to synthesize them.

Plant secondary metabolites are typically produced in response to changes in the environment or pathogen/herbivore attack and are currently in the spotlight of biotechnology because of their applications in different industrial sectors (i.e., healthcare, cosmetics, to name a few; Guerriero et al., 2018). The chemical structures of plant functional molecules are able to counteract reactive oxygen species (ROS) and reactive nitrogen species (RNS) by acting as natural scavengers (Berni et al., 2018b). A strong body of evidence in the literature has indeed drawn attention on plant secondary metabolites in the light of their antioxidant potential, by focusing on specific classes, namely terpenes, and polyphenols (Ghasemzadeh et al., 2010; Thoppil and Bishayee, 2011).

Terpenes consist of over 40,000 different molecules produced by many organisms, spanning from bacteria, yeasts, fungi and, most notably, plants (Goto et al., 2010). They have been studied for their anti-inflammatory effects (Hortelano, 2009) linked to the regulation of cytokine production in human cells (Ku and Lin, 2013). In the plant kingdom, these molecules are synthesized in large quantities by food crops, such as tomatoes: these vegetables produce large amounts of terpenes in the form of carotenoids and, in particular, of lycopene, a pigment that is responsible for the typical red color (Saini et al., 2015). Lycopene is the most efficient quencher of free radicals with the property of protecting the cellular components against oxidative damage (Nasir et al., 2015).

Polyphenols are the largest group of plant secondary metabolites and are classified into different subsets of compounds, depending on their chemistry (Tsao, 2010; Kabera et al., 2014). Their structure is characterized by aromatic ring(s) linked to hydroxyl groups, which confer antioxidant properties to the molecule (Craft et al., 2012). Particular scientific attention is devoted to the class of flavonoids that are present in common plant food products. Vegetables, such as onions, contain high levels of flavonoids, notably quercetin, that is well known for its applications in cancer prevention (Gibellini et al., 2011). Furthermore, evidence in disease treatment is reported for other flavonoids, such as anthocyanins (Norberto et al., 2013). The role of these molecules is directly linked to the decreased susceptibility in developing cardiovascular diseases (Wallace, 2011).

Red fruits like sweet cherries typically contain high levels of anthocyanins and their dietary intake could help in the prevention of cardiovascular diseases (He and Giusti, 2010; Mirto et al., 2018).

Fruits and vegetables are thus natural sources of different bioactives: in the light of their documented benefit on human health, their intake through the daily diet contributes to boost the natural body defenses.

We have here focused on three examples of crops/fruit tree, i.e., tomatoes, onions, and sweet cherry, which are constituents of the Mediterranean diet and are part of the rich biodiversity of Italian regions. In Tuscany, which is the case study here analyzed, 8 different tomato varieties were previously reported (Naziri, 2009) and specific onion varieties are included in the “PAT” (Prodotti alimentari tradizionali-Traditional food products) by the Ministry of Agricultural Politics (Ferioli and D'Antuono, 2016). The fruits/bulbs of these species are not only consumed fresh, but also dried, or processed as soups/juice/jams/marmalades and canned foods. Additionally, tomatoes and onions are produced for >0,6 million tons and >300,000 tons per year in Italy (statistics from 2010; Sardaro et al., 2013; Caruso et al., 2014) and therefore represent economically-important crops.

## Native (Locally-Grown) Plants vs. Commercial Plants

Native crops are wild plants that are consumed for their fruits, leaves, flowers, and seeds and originating in specific geographic areas (Glew et al., 2005). The concept of native crops can be extended to comprise also plants cultivated for several decades in a specific territory and not necessarily originating from the same territory. Native or wild plants have adapted to the territory by establishing a synergy with the local soil and climate. They represent the local germplasm and offer a source of genes and unique phenotypic characters (Berni et al., 2018a). Emblematic is the example of non-commercial apple varieties showing extreme phenotypes, notably russeting (Legay et al., 2015, 2017) and containing molecules with immune-modulatory properties (Andre et al., 2013).

The inexorable industrial development has selected varieties of fruit and vegetables according to the market demand. Varieties that are not productive according to commercial standards are put aside and this leads, indirectly, to a loss of biodiversity (Schmidt and Wei, 2006).

For centuries, wild crops have played a significant role in nutrition, especially during times of weather extremes, thanks to their ability to withstand strong exogenous stresses, thereby representing a key feedstock for human nutrition (Bvenura and Afolayan, 2015). Furthermore, the fruits of such ancient varieties produce a wide range of bioactive molecules that are currently studied for their role in the human diet (Berni et al., 2018c) and as protective molecules against diseases (Francini et al., 2017; Berni et al., 2018b).

The adaptive strategies put in place by these plants to survive in a wild environment (not impacted by any human intervention) translate into an enhanced production of secondary metabolites, such as polyphenols and terpenes. The increased production of such molecules is likely linked to epigenetic changes that have been induced by the interaction with the environment (Baulcombe and Dean, 2014). Genetic modifications confer to ancient crops unique phenotypic and nutritional features, compared to fruits cultivated for commercial purposes (Legay et al., 2017). In this sense, many authors have focused their attention on the functional molecules derived from autochthonous fruits to shed light on their nutritional and nutraceutical potential in the human diet (Stintzing and Carle, 2004). Ancient fruits are considered as natural antioxidant resources and potential scavengers against oxidative stress. Very interesting, in this respect, are the studies that compare the content of these molecules, expressed in terms of antioxidant potential, in ancient and commercial species. Iacopini et al. (2010) report that the ancient apple varieties they studied contain a higher concentration of antioxidant molecules, with respect to commercial counterparts. We have also recently shown that Tuscan tomatoes sampled in 2016 show higher contents of polyphenols and antioxidant molecules with respect to commercial varieties and that this increase is two- and even three-fold (Berni et al., 2018c). As discussed by other authors, the genotype plays a fundamental role on the final content of functional molecules in fruits (Scalzo et al., 2005), since its interaction with the surrounding environment determines the expression of specific traits (King, 2015). Researches on autochthonous fruits intend to valorize and promote the preservation of these territorial species, by exploiting their nutritional value (Lamien-Meda et al., 2008). Considering the huge spectrum of functional molecules produced, ancient crops can, and should be considered as functional foods and could even be used in support of drug therapies.

## The Case Study of Native Crops in Tuscany (Italy): Tomatoes, Sweet Cherries and Onions

The region of Tuscany is world renowned as a producer of high-quality products, such as wine and oil. This unrivaled product quality is due to the local production and exclusive use of territorial natural resources (Mangani et al., 2011), as well as to the protection of specific cultivars (Berni et al., 2018a) and to the development of quality labels. The growing interest in these foods is fuelled by their high content in health-beneficial bioactive molecules (Iacopini et al., 2008; Cavallini et al., 2014). The rich composition in bioactives has the potential of making these products key components of a functional diet (Cencic and Chingwaru, 2010) that is able to offer a stronger line of defense against diseases (Pandey and Rizvi, 2009). Thanks to the ability to adapt to the territory, these plants have developed a synergy with the local soil. In this sense, Tuscany has a broad panoply of plant species that constitute the regional germplasm heritage (http://germoplasma.regione.toscana.it/index.php?option=com_content&view=article&id=1&Itemid=127). Most of these plants were commonly used in the past for human consumption or for other uses: fruit harvesting, wood and fodder, to mark the borders and to support other plants and wild animals. Several evidences in the literature indicate that in these woody and herbaceous species the fruits show noteworthy contents of nutritional and functional compounds (Ancillotti et al., 2016). Therefore, analytical studies are necessary to highlight the functional properties of these local species expressed in terms of bioactive compound contents. The case study here reported will shed light on the nutraceutical characteristics of ancient varieties of Tuscan onions, tomatoes and sweet cherries to sensitize the public to a wider use of these varieties that play an important role in the regional biodiversity landscape and that could ultimately improve human nutrition.

Tomatoes are well known for their content in carotenoids, such as lycopene, but also for the occurrence of other molecules, notably flavonoids and hydroxycinnamic acids (García-Valverde et al., 2013). Onions are an excellent source of flavonoids, i.e., myricetin, quercetin and kaempferol. These molecules are typically present in high amount in the fruit flesh; on the contrary, the skins of red onions are rich in anthocyanins (Pérez-Gregorio et al., 2010). Sweet cherries are one of the most abundant red fruits rich in anthocyanins, distributed chiefly in the peel and in the outer layers of the fruit (Kelebek and Selli, 2011). Nevertheless, in some varieties of these fruits, the distribution of anthocyanins is also found in the seeds. Tuscany has classified 8 varieties of tomatoes, 6 of onions and 6 of sweet cherries; these crops are grown in experimental fields (http://www.ivalsa.cnr.it/az-s-paolina.html), which are used to preserve the native genetic resources located in various areas of the region.

**Figure 1:**
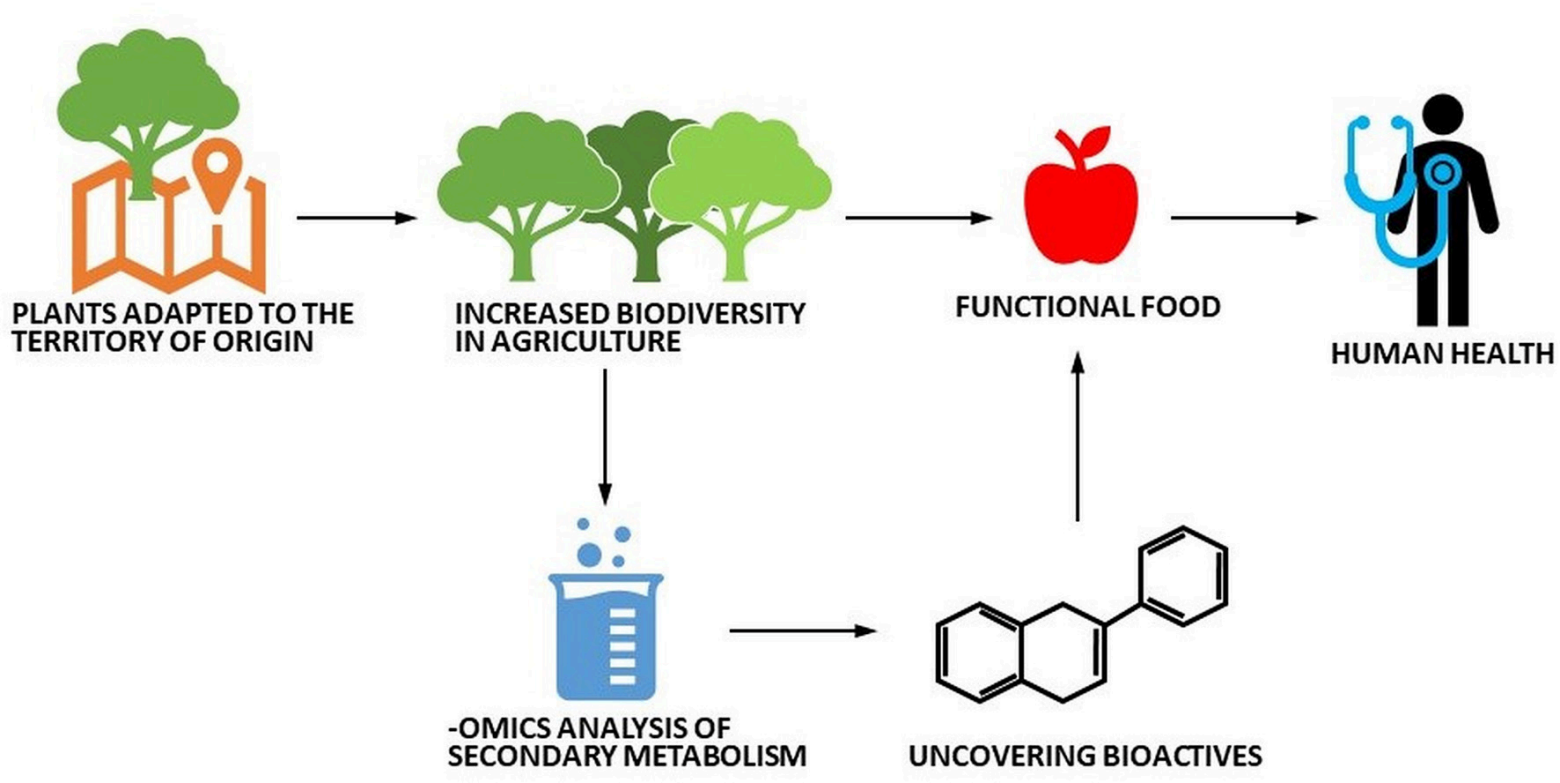
Schematic cartoon depicting the potential valorization route of local ancient plant varieties.

The plant material used in the present study was provided by the “Trees and Timber Institute—CNR-IVALSA” (Follonica, Italy). The institute is a germplasm regional bank for the propagation and enhancement of regional genetic resources. Our analysis focused on functional molecules in native varieties collected in 2017, with the aim of comparing their content to that found in the most widespread commercial Italian varieties. All the analyses performed follow the standards used for the determination of functional and nutraceutical molecules, i.e., Ferric Reducing Antioxidant Power (FRAP), Folin–Ciocalteu, aluminum chloride assay, pH differential method (Aramwit et al., 2010; Dai and Mumper, 2010; Jagtap et al., 2010) and confirmed with HPLC analysis (see Supplementary Material for a description of the methods used). The results are reported in Table 1. Regional varieties showed interesting results, especially when compared with commercial counterparts. Autochthonous fruits contained indeed large quantities of functional molecules (polyphenols, flavonoids, and terpenes) that, in many cases, were higher than those found in commercial fruits (Berni et al., 2018c). Notably, a higher functional molecule content has been demonstrated in other crop landraces (Renna et al., 2014, 2018). These results show that local fruits produce higher amounts of molecules that can be exploited as health-beneficial bioactive compounds (Williamson, 2017; Figure 1).

**Table 1:** Total content of antioxidants as mmol Fe^2+^ per 100g FW, polyphenols as mg of GAE (gallic acid equivalents) per 100 g of FW, flavonoids as mg of QeE (quercetin equivalents) per 100 g of FW, carotenoids as TCC (total carotenoids content) per 100 g of FW and anthocyanins as CyE (cyanidin-3-glucoside equivalents) per 100g of FW.

**Variety names**	**Antioxidants (mmol Fe^**2+**^/100 g FW) ± S.D**	**Polyphenols (mg GAE/100g FW) ± S.D**	**Flavonoids (mg QeE/100g FW) ± S.D**	**Carotenoids (mg TCC/100g FW) ± S.D**	**Anthocyanins (mg CyE/100g FW) ± S.D**
**Tomatoes (*****Solanum lycopersicum*** **L.)**					
Liscio da Serbo Toscano	0.84 ± 0.04a	74.24 ± 1.7a	12.96 ± 0.2ae	0.80 ± 0.02a	/	
Rosso Pitigliano	1.19 ± 0.01b	75.67 ± 1.7a	7.25 ± 0.1b	0.86 ± 0.06ab	/	
Quarantino ec Valdarno	1.03 ± 0.02c	90.35 ± 1.1b	20.31 ± 0.5c	0.90 ± 0.04b	/	
Fragola	0.64 ± 0.03d	63.68 ± 1.2c	8.83 ± 0.1d	0.87 ± 0.03ab	/	
Canestrino di Lucca	0.52 ± 0.04e	53.81 ± 2.5d	7.88 ± 0.1b	0.89 ± 0.01ab	/	
Costoluto Fiorentino	1.14 ± 0.03b	76.78 ± 2.4ae	13.42 ± 0.2e	0.75 ± 0.05c	/	
Giallo di Pitigliano	0.93 ± 0.02a	79.24 ± 0.7e	21.49 ± 0.4c	0.71 ± 0.07c	/	
Pisanello	0.76 ± 0.02f	66.52 ± 0.4c	11.95 ± 0.2a	0.74 ± 0.01c	/	
Cuore di Bue (Commercial)	0.48 ± 0.07e	51.35 ± 1.5d	6.58 ± 0.1f	0.69 ± 0.07c	/	
**Onions (*****Allium cepa*** **L.)**					
Maremma	1.84 ± 0.10a	69.25 ± 2.6a	63.2 ± 1.3a	/	7.1 ± 0.8a	
Rossa Massese	3.08 ± 0.14b	302.83 ± 3.9b	280.4 ± 3.2b	/	40.2 ± 2.1b	
Treschietto	1.18 ± 0.08cde	150.42 ± 4.9c	69.3 ± 1.4c	/	6.8 ± 0.6a	
Rossa della Valtiberina	1.15 ± 0.11cd	98.58 ± 3.1d	68.3 ± 3.5c	/	55.5 ± 2.8c	
Rossa di Lucca	1.23 ± 0.12d	117.91 ± 4.2e	80.2 ± 2.1d	/	23.3 ± 1.8d	
Rossa Fiorentina	0.97 ± 0.09e	89.52 ± 2.9f	60.3 ± 2.9a	/	47.3 ± 2.3b	
Tropea (Commercial 1)	0.91 ± 0.02e	55.98 ± 0.9g	24.4 ± 0.9e	/	39.5 ± 2.4b	
Giarratana (Commercial 2)	0.83 ± 0.01e	58.76 ± 4.7g	27.9 ± 0.8e	/	/	
**Sweet cherries (*****Prunus avium*** **L.)**					
Carlotta	1.49 ± 0.06a	200.49 ± 15.8a	47.21 ± 2.4a	/	30.80 ± 1.8a	
Benedetta	1.51 ± 0.04a	250.91 ± 11.3b	65.33 ± 2.3b	/	27.52 ± 1.8a	
Morellona	2.51 ± 0.03b	300.87 ± 13.1c	63.01 ± 3.7b	/	59.41 ± 2.5b	
Maggiola	1.92 ± 0.12c	276.71 ± 7.5d	46.41 ± 2.5a	/	33.17 ± 1.7a	
Moscatella	1.51 ± 0.05a	215.15 ± 11.9a	44.82 ± 4.8a	/	27.04 ± 2.4ca	
Crognola	3.17 ± 0.01d	368.18 ± 5.7e	81.62 ± 3.3c	/	67.75 ± 2.7c	
Durone (Commercial)	1.13 ± 0.04e	146.05 ± 2.7f	32.21 ± 2.8d	/	31.65 ± 2.3a	
**Tomatoes (*****Solanum lycopersicum*** **L.)**	**Caffeic Acid (μg/g FW) ± S.D**	**Ferulic Acid (μg/g FW) ± S.D**	**Chlorogenic Acid (μg/g FW) ± S.D**	**Naringenin (μg/g FW) ± S.D**	**Quercetin (μg/g FW) ± S.D**	**Lycopene (μg/g FW) ± S.D**
Liscio da Serbo Toscano	18.75 ± 0.9a	3.89 ± 0.5a	41.23 ± 2.4a	5.48 ± 0.9ad	14.08 ± 2.6a	4.65 ± 0.3a
Rosso Pitigliano	17.21 ± 1.4a	8.25 ± 1.2b	28.26 ± 1.8b	14.24 ± 2.1b	27.45 ± 3.4b	4.23 ± 0.1a
Quarantino ec Valdarno	9.50 ± 0.8b	8.56 ± 1.4b	39.56 ± 2.9a	4.01 ± 0.7a	20.14 ± 2.9c	6.12 ± 0.5b
Fragola	7.45 ± 2.1c	7.81 ± 0.9b	20.14 ± 1.7c	19.12 ± 1.4c	18.01 ± 1.7c	5.06 ± 0.3a
Canestrino di Lucca	8.14 ± 0.8b	3.01 ± 1.2a	19.84 ± 1.9c	6.57 ± 1.2ad	17.12 ± 2.7c	5.89 ± 0.6ab
Costoluto Fiorentino	14.56 ± 1.7d	8.12 ± 1.7b	30.21 ± 2.1b	6.14 ± 0.9ad	9.87 ± 1.5d	4.15 ± 0.2a
Giallo di Pitigliano	8.23 ± 1.2b	9.16 ± 1.9b	18.54 ± 0.7c	8.14 ± 1.9d	10.48 ± 2.7d	4.58 ± 0.5a
Pisanello	19.54 ± 1.4a	2.28 ± 0.9a	16.02 ± 2.4c	3.89 ± 0.4a	10.47 ± 2.8d	6.21 ± 0.7b
Cuore di Bue (Commercial)	5.14 ± 1.2e	0.98 ± 0.8c	13.24 ± 1.8d	2.01 ± 0.2e	6.48 ± 0.7e	4.02 ± 0.1a
**Onions (*****Allium cepa*** **L.)**	**Quercetin (μg/g FW) ± S.D**	**Myricetin (μg/g FW) ± S.D**	**Kaempferol (μg/g FW) ± S.D**	**Peonidin-3-glucoside (μg/g FW) ± S.D**	**Petunidin-3-glucoside (μg/g FW) ± S.D**	
Maremma	29.59 ± 0.6a	30.59 ± 7.6a	216.58 ± 8.4a	9.12 ± 0.4a	5.12 ± 1.3a	
Rossa Massese	583.77 ± 3.1b	583.77 ± 10.2b	849.14 ± 10.4b	21.64 ± 2.3b	15.24 ± 1.4b	
Treschietto	23.54 ± 0.7c	23.54 ± 8.1a	305.68 ± 7.8c	9.57 ± 0.8a	4.91 ± 1.2a	
Rossa della Valtiberina	24.26 ± 0.9c	24.26 ± 9.6a	304.45 ± 8.7c	36.47 ± 1.9c	21.45 ± 1.3c	
Rossa di Lucca	183.12 ± 2.1d	183.12 ± 8.2c	251.06 ± 8.1d	10.57 ± 1.1a	7.15 ± 1.9ad	
Rossa Fiorentina	22.67 ± 1.2c	22.67 ± 6.4a	221.08 ± 8.9a	31.18 ± 0.7d	20.14 ± 2.1c	
Tropea (Commercial 1)	19.86 ± 2.3c	19.86 ± 8.1a	66.68 ± 2.4e	10.96 ± 0.5a	10.24 ± 2.7d	
Giarratana (Commercial 2)	20.45 ± 2.5c	20.14 ± 7.4a	87.78 ± 5.6f	/	/	
**Sweet cherries (*****Prunus avium*** **L.)**	**Chlorogenic Acid (μg/g FW) ± SD**	***p*****-coumaric acid (μg/g FW) ± SD**	**(+)-Catechin (μg/g FW) ± SD**	**Rutin (μg/g FW) ± SD**	**Cyanidin-3-glucoside (μg/g FW) ± SD**	
Carlotta	121.91 ± 0.6a	19.41 ± 02a	25.93 ± 1.9a	30.83 ± 1.3a	51.78 ± 1.8a	
Benedetta	81.27 ± 0.4b	16.61 ± 0.3b	201.54 ± 1.4b	53.96 ± 3.4b	33.88 ± 1.5b	
Morellona	324.59 ± 0.4c	30.05 ± 0.2c	72.54 ± 2.3c	38.54 ± 1.4c	74.82 ± 1.1c	
Maggiola	94.32 ± 0.2d	12.43 ± 0.2d	45.99 ± 1.9d	34.46 ± 1.8c	35.28 ± 1.3b	
Moscatella	239.25 ± 01e	10.13 ± 0.3e	34.86 ± 2.7e	27.93 ± 1.6a	33.54 ± 1.6b	
Crognola	387.73 ± 07f	28.11 ± 0.3f	163.51 ± 1.4f	95.33 ± 2.1d	151.2 ± 1.2d	
Durone (Commercial)	78.25 ± 0.4b	10.85 ± 0.1e	15.85 ± 1.1g	25.65 ± 2.1a	36.27 ± 1.7b	

*The table also reports the HPLC quantifications of specific secondary metabolites in tomatoes, onions and sweet cherries expressed as μg of component per gram of FW. Values are indicated with the relative standard deviation; different letters refer to statistically significant differences (p < 0.05)*.

The use of these fruits could represent the next step towards the diversification of the current staple food crop market and a functional diet valorizing the local agrobiodiversity (Cantini et al., 2018). Additionally, such varieties could be the object of high-throughput studies based on—*omics* to uncover the bioactive molecules produced and to test their nutraceutical and health-promoting effects (Figure 1).

## Is the Cultivation of Local Plants An Economic Resource?

Assuming the importance to take functional molecules with the diet, there are contrasting aspects that sometimes make vain the scientific interest on these molecules. One example is the input for the local population to consume local products in opposition to mass-merchandised products, which is often more a cultural than an economic issue. The consumption of products from the local agriculture might be perceived as a return to “old-fashioned” agriculture that is considered less productive and less profitable. Therefore, the consumption of local products is often seen by consumers as the return to an “ancient” agriculture. This may be more a problem for younger generations than older ones. The new generations have had different cultural backgrounds in which nutrition was not seen as a way to improve health, but essentially as a way to simply take on calories (Popkin et al., 2012). Not going too far into the basic rhetoric about fast-food, this different perception has greatly contributed to affect the trends in agriculture. The consumption of products from the local agriculture also necessarily requires the cultivation of local plants on a large scale. The problem then shifts from the consumer to the farmer and the question is: how ready are farmers to grow local plants instead of more traditional and therefore more commercial ones? This is a very serious question because it implies rethinking on the production yield of these plants. Obviously, no farmer prefers to earn less and it is therefore clear that locally adapted plants must be as productive in economic terms as more traditional ones (Reganold and Wachter, 2016). So far, few researches have been focused on the differences in biodiversity and on the economic benefits between unconventional and conventional crops.

Tuscany has been particularly active in recent years by promoting research and transfer activities in the agro-food sector (Berni et al., 2018a). Through a series of financially-supported projects, Tuscany has actively promoted and supported the research on and the cultivation of autochthonous species rich in functional molecules. An emblematic output of these projects is the manufacture of dark chocolate bars (Toscolata®) functionalized with Tuscan autochthonous food products (Cantini et al., 2018).

In particular, we hereby wish to mention two projects to which we have recently participated: the first concerns the genetic, qualitative and sustainability characterization of products and derivatives from autochthonous Tuscan horticultural crops (BASIQ project: http://www.valdimersegreen.com/basiq/). The second project is about the use of different cultivation practices of wheat to see how it might affect the content of bioactive molecules (INNOVACEREALI project: http://innovacereali.maidicolasovicille.it/).

## The Cultivation of Native Plants Preserves the Local Agrobiodiversity

This Perspective paper on the importance of cultivating native species, albeit limited to a specific region of Central Italy, can be enriched by one further final consideration: the cultivation of native plants cannot only preserve the local agrobiodiversity (Berni et al., 2018a), but also contribute to the restoration of original regional habitats. Nowadays, the loss of habitat diversification is a major threat linked to the ever-growing industrialization, climatic changes and land-use. Future forecasts highlight the dramatic scenarios of habitat loss with the increasing use of wild lands for the massive cultivation of man-selected plants (Pereira et al., 2010). Landscape and plant biodiversity are directly linked: the loss of one involves the loss of the other. The cultivation of native crops can play an important role in the preservation of both, while more traditional agricultural practices, especially if intense, can have negative consequences because of the massive use of land resources to promote large-scale products. Therefore, a well-conceived agricultural management, based on the regional valorization of autochthonous species, has been shown to preserve biodiversity and local ecosystems (Tscharntke et al., 2005). Native plants are also useful to maintain the local soil microbiota and composition, thereby preserving an optimal interaction between plants and microorganisms. Lange and colleagues reported the importance of plant diversity in the microbial soil composition with indirect effects also on C storage of the whole ecosystem (Lange et al., 2015).

Based on these evidences, the use of native crops should not aim at massive agricultural production, but rather at maintaining and restoring habitats in regional “niches.” From this perspective, the cultivation of local plants has both an economically- and ecologically-relevant impact.

## Author Contributions

RB, GG, and MR wrote the manuscript and prepared the figures. J-FH, CC, and GC revised the text.

### Conflict of Interest Statement

The authors declare that the research was conducted in the absence of any commercial or financial relationships that could be construed as a potential conflict of interest.
