# Supplementary material for: Functional Molecules in Locally-Adapted Crops: The Case Study of Tomatoes, Onions, and Sweet Cherry Fruits From Tuscany in Italy
Source: Front Plant Sci. 2019 Jan 15;9:1983. doi: 10.3389/fpls.2018.01983 (PMC6341061; doi:10.3389/fpls.2018.01983)
Supplement: Supplementary file 1 [file Data_Sheet_1.docx]

**Supplementary Information**

**Materials and methods**

Plants were grown in an experimental field under the administration of the National Research Council of Italy (CNR IVALSA), located in Follonica (Italy) (42°55'59.7"N 10°45'53.0"E). Sweet cherries, onions and tomatoes WERE harvested in 2017 at the same level of ripeness. Fruits were harvested in the experimental field and immediately placed at -80° C to block any metabolic process.

Onions and tomatoes were analyzed in three independent biological replicas, as well as three experimental replicates for each variety.

Cherries were analyzed in 8-10 biological replicates and three experimental replicates for each variety.

**Sample extraction**

For antioxidants, polyphenols and flavonoids, the extraction procedure was carried out following the method described by Henríquez et al., (2010). Samples were extracted in 70% acetone (1:3 w/v).

For anthocyanins, the extraction procedure is described in Hosu et al., (2014). Anthocyanins were extracted with acidified ethanol (0.1% HCl 1:1 w/v).

Carotenoids were extracted using a solvent composed of methanol, acetone, hexane 50:25:25, 1:1 w/v (Berni et al., 2018c).

**Chemical assays**

**Antioxidant capacity**

FRAP (Ferric Reducing Antioxidant Power) was used as antioxidant capacity assay (Benzie and Strain, 1996). The absorbance values at 593 nm were obtained by comparing the samples with a standard curve (solutions of ferric chloride standard at different concentrations) and reported as mmol of ferric chloride (Fe**^2+^**) equivalent per 100 grams of fresh weight (FW).

**Phenolic contents**

The Folin-Ciocalteau method was used as phenolic quantification assay (Singleton and Rossi, 1965). Sample values were compared with a standard curve (solutions at different concentrations of gallic acid standard) and expressed as gallic acid equivalents (GAE) per 100 grams of FW. The absorbance was set at 765 nm.

**Flavonoid contents**

The aluminum chloride method was used for flavonoid determination, following the method described in Ebrahimzadeh et al., (2008). Results were expressed as milligrams of quercetin equivalents (QeE) per 100 grams of fruit FW, comparing the absorbance (set at 415 nm) of the samples with a standard curve (quercetin standard concentrations).

**Anthocyanin contents**

Total anthocyanins were determined according to the pH differential spectroscopic method (Tonutare et al., 2014). Samples were measured at different absorbance values (510, 700 nm) and the following mathematical formulas were applied (Tonutare et al., 2014).

A_sp_ = (A510 – A700) pH 1.0 – (A510 – A700) pH 4.5

Total anthocyanins (TA) = (Asp × M ×DF × 1000) / (ɛ× λ × m)

Asp= spectrophotometrically measured absorption values M= molecular weight, DF= dilution factor, ɛ= molar absorptivity coefficient, λ= cuvette optical path-length (1 cm), m= weight of the sample (g).

The total anthocyanin content was expressed as mg of cyanidin-3-glucoside equivalents (CyE) per 100 g of FW.

**Evaluation of carotenoid contents**

The carotenoid content was evaluated as previously described by Lichtenthaler and Wellburn, (1983). Three different wavelengths were used (662, 645, 470 nm) and the following mathematical formulas reported by Lichtenthaler and Wellburn, (1983) were applied:

C_a_ = 1 1.75A_662_-2.35A_645_

C_b_ = 18.61A_645_-3.96A_66_

C_x+c_ = (1000A_470_-2.27Ca-81.4 Cb) /227

C_a_= Contents of chlorophyll a, C_b_= contents of chlorophyll b, C_x+c_= contents of carotenoids.

Total carotenoid contents (TCC) were expressed as mg per 100 g of FW.

**HPLC assays**

**Polyphenols and Flavonoids**

Polyphenols were extracted using the method described in Tokuşoğlu et al., (2003) (1:1 w/v). The HPLC method was based on a previous work (Kumar et al., 2008). Results were reported as µg of molecule per g of FW, comparing the resulting chromatograms with standard curves (at the increasing concentrations of 0.5, 2, 5, 12, 25, 50 μg/ml) of selected standard molecules (caffeic acid, ferulic acid, chlorogenic acid, *p*-coumaric acid, naringenin, quercetin, myricetin, kaempferol, (+)-catechin).

**Anthocyanins**

Anthocyanins were prepared for HPLC analysis following the method described by Nyman and Kumpulainen, (2001) (1:1 w/v. The HPLC analysis was performed following the method previously reported in Luczkiewicz and Cisowski, (1998). Results were compared with pre-built standard curves (at the increasing concentrations of 0.5, 2, 5, 12, 25, 50 μg per ml) of standard reagents (cyanidin-3-glucoside, peonidin-3-glucoside, petunidin-3-glucoside) and reported as μg of molecules per g of FW.

**Lycopene**

Lycopene was extracted performing the method described by Barba et al., (2006) (1:1 w/v). The HPLC analysis was carried out following a previously described method (Berni et al., 2018c) comparing the samples with a standard curve composed by different concentrations of lycopene standard (at the increasing concentrations of 0.5, 2, 5, 12, 25, 50 μg).

**Supplementary** **references**

Barba, A. O., Hurtado, M. C., Mata, M. S., Ruiz, V. F., and De Tejada, M. L. S. (2006). Application of a UV–vis detection-HPLC method for a rapid determination of lycopene and β-carotene in vegetables. *Food Chem.* 95, 328–336.

Benzie, I. F., and Strain, J. J. (1996). The ferric reducing ability of plasma (FRAP) as a measure of “antioxidant power”: the FRAP assay. *Anal. Biochem.* 239, 70–76.

Ebrahimzadeh, M. A., Pourmorad, F., and Hafezi, S. (2008). Antioxidant activities of Iranian corn silk. *Turk. J. Biol.* 32, 43–49.

Henríquez, C., Almonacid, S., Chiffelle, I., Valenzuela, T., Araya, M., Cabezas, L., et al. (2010). Determination of antioxidant capacity, total phenolic content and mineral composition of different fruit tissue of five apple cultivars grown in Chile. *Chil. J. Agric. Res.* 70, 523–536.

Hosu, A., Cristea, V.-M., and Cimpoiu, C. (2014). Analysis of total phenolic, flavonoids, anthocyanins and tannins content in Romanian red wines: Prediction of antioxidant activities and classification of wines using artificial neural networks. *Food Chem.* 150, 113–118.

Kumar, N., Bhandari, P., Singh, B., Gupta, A. P., and Kaul, V. K. (2008). Reversed phase-HPLC for rapid determination of polyphenols in flowers of rose species. *J. Sep. Sci.* 31, 262–267.

Lichtenthaler, H. K., and Wellburn, A. R. (1983). Determinations of total carotenoids and chlorophylls a and b of leaf extracts in different solvents. Portland Press Limited.

Luczkiewicz, M., and Cisowski, W. (1998). The RP-HPLC analysis of anthocyanins. *Chromatographia* 48, 360–364.

Nyman, N. A., and Kumpulainen, J. T. (2001). Determination of anthocyanidins in berries and red wine by high-performance liquid chromatography. *J. Agric. Food Chem.* 49, 4183–4187.

Singleton, V. L., and Rossi, J. A. (1965). Colorimetry of total phenolics with phosphomolybdic-phosphotungstic acid reagents. *Am. J. Enol. Vitic.* 16, 144–158.

Tokuşoğlu, Ö., Ünal, M. K., and Yıldırım, Z. (2003). HPLC-UV and GC-MS characterization of the flavonol aglycons quercetin, kaempferol, and myricetin in tomato pastes and other tomato-based products. *Acta Chromatogr* 13, 196–207.

Tonutare, T., Moor, U., and Szajdak, L. (2014). Strawberry anthocyanin determination by ph differential spectroscopic method – how to get true results? 13.
